# Effects of LED Light Combinations on the Growth and Storage Stability of *Ipomoea aquatica* in a Plant Factory System

**DOI:** 10.3390/plants15050776

**Published:** 2026-03-03

**Authors:** Si-Hong Kim, Jeong-Eun Sim, Ah-Young Shin, Yu-Jin Kang, Han-Kyeol Park, Jae-Kyung Kim, Ju-Yeon Ahn, Byeong-Jun Jeon, Ho-Min Kang

**Affiliations:** 1 Smart Farm Research Center, KIST Gangneung, Institute of National Products, 679 Saimdang-ro, Gangneung 25451, Republic of Korea; tlghdek@kist.re.kr; 2 Interdisciplinary Program in Smart Agriculture, Kangwon National University, Chuncheon 24341, Republic of Korea; jamma2659@kangwon.ac.kr (J.-E.S.); tlsdkdud603_@kangwon.ac.kr (A.-Y.S.); kk09066@naver.com (Y.-J.K.); fallphk@kangwon.ac.kr (H.-K.P.); 3 Department of Smartfarm, Chungbuk Provincial University, Okcheon 29046, Republic of Korea; jaekim@cpu.ac.kr; 4 Gyeonggido Agriculture Research & Extension Services, Hwaseong 18388, Republic of Korea; jyahn0926@gg.go.kr; 5 Department of Plant Science, Kangwon National University, Gangneung 25457, Republic of Korea

**Keywords:** light quality, microbial safety, modified atmosphere packaging (MAP), photosystem II efficiency, plant factory with artificial lighting (PFAL)

## Abstract

This study investigated how different LED spectral compositions affect seed germination, early growth, photosynthetic efficiency, and the postharvest quality and microbiological stability of *Ipomoea aquatica* Forsk. cultivated in a plant factory system, aiming to propose an integrated management strategy for stable year-round production. Five LED light treatments with varying red and blue light ratios (R10, R7B3, R5B5, R3B7, and B10) were applied during cultivation. After harvest, the plants were stored under low-temperature conditions using either carton box packaging or modified atmosphere packaging (MAP) to evaluate postharvest quality and microbial changes. Germination analysis indicated that red-dominant treatments (R10 and R7B3) significantly enhanced germination percentage, rate, and uniformity. These treatments also promoted greater plant height and fresh biomass accumulation during early growth while maintaining a higher maximum quantum yield of photosystem II (*Fv*/*Fm*), indicating improved photochemical efficiency. In contrast, blue-dominant treatments led to reduced growth performance and lower *Fv*/*Fm* values. Postharvest quality and microbiological stability were more significantly affected by the packaging method than by the LED light treatment. MAP effectively minimized fresh weight loss and inhibited the growth of aerobic bacteria, *Escherichia coli*, total coliforms, and yeast and mold during storage. Overall, the findings demonstrate that red-centered LED spectra are optimal for enhancing early growth and physiological stability of *I. aquatica*, while MAP is crucial for preserving postharvest quality and microbial safety. This study underscores the synergistic potential of combining LED spectral management during cultivation with optimized packaging strategies to achieve stable year-round production and extended shelf life of *I. aquatica* in controlled plant factory systems.

## 1. Introduction

In recent years, a growing global interest in health promotion and preventive medicine has underscored the significance of dietary intake of functional bioactive compounds. Plant-derived functional constituents, in particular, have been shown to possess a wide range of physiological activities, such as antioxidant, anti-inflammatory, antimicrobial, antidiabetic, and anti-obesity effects. This has led to an increase in both the consumption and research of functional vegetables and medicinal plants. Within this context, leafy vegetables are highly regarded as valuable functional food sources in daily diets because they are rich in vitamins, minerals, polyphenols, and dietary fiber [1,2].

*Ipomoea aquatica* Forsk, commonly known as water spinach, is a leafy vegetable from the Convolvulaceae family, traditionally consumed as both food and medicine in Southeast Asia and tropical to subtropical regions. This crop contains various bioactive compounds, including vitamins A and C, iron, calcium, flavonoids, phenolic compounds, and carotenoids. It has been reported to exhibit antioxidant and anti-inflammatory activities, as well as beneficial effects on blood glucose regulation, liver protection, and immune function [3]. However, due to its tropical origin and vigorous growth in hot and humid conditions, *I. aquatica* often demonstrates unstable growth when cultivated in domestic environments. Additionally, its high water content in leaves and stems limits postharvest storability and marketability, posing challenges for storage and distribution [4].

To address the limitations in cultivation and postharvest handling, fully controlled plant factory systems with artificial lighting (PFALs) have gained increasing attention in modern horticultural production. These systems reduce the influence of external environmental fluctuations and enable precise regulation of light, temperature, humidity, and carbon dioxide concentration, allowing year-round production of crops with consistent quality. Recent advances in PFAL technology have emphasized spectral optimization strategies to enhance productivity and resource-use efficiency [5]. Among artificial light sources, light-emitting diodes (LEDs) are particularly suitable for PFAL systems because of their high energy efficiency and ability to emit specific wavelengths. Red and blue light are considered the most physiologically effective regions of the photosynthetically active radiation spectrum. Red light primarily promotes photosynthetic activity and shoot elongation through phytochrome-mediated pathways, whereas blue light regulates chlorophyll synthesis, stomatal function, and photomorphogenesis via cryptochrome- and phototropin-mediated signaling. Importantly, studies on leafy vegetables such as lettuce, spinach, and microgreens have demonstrated that the ratio of red to blue light significantly affects plant morphology, biomass accumulation, and nutritional quality [6,7,8]. Red-dominant spectra generally enhance shoot growth and fresh weight, while higher blue-light proportions improve compact morphology and physiological balance. Therefore, optimizing the red-to-blue light ratio is regarded as a key strategy for improving growth efficiency and product quality in plant factory systems.

Numerous studies have examined the effects of LED light quality on germination and growth in crops such as lettuce, spinach, microgreens, and various medicinal plants [9,10]. However, research on the effects of LED spectral quality on *I. aquatica* remains limited. Specifically, comprehensive analyses of germination characteristics, growth performance, and physiological responses under plant factory conditions are scarce. Thus, this study aims to investigate the impact of different red and blue LED light combinations on the germination behavior, growth characteristics, and physiological responses of *I. aquatica* cultivated in a plant factory system, providing essential information for achieving stable year-round production of this crop.

## 2. Results

### 2.1. Effects of Light Quality on Seed Germination of Ipomoea aquatica

**Figure 1:**
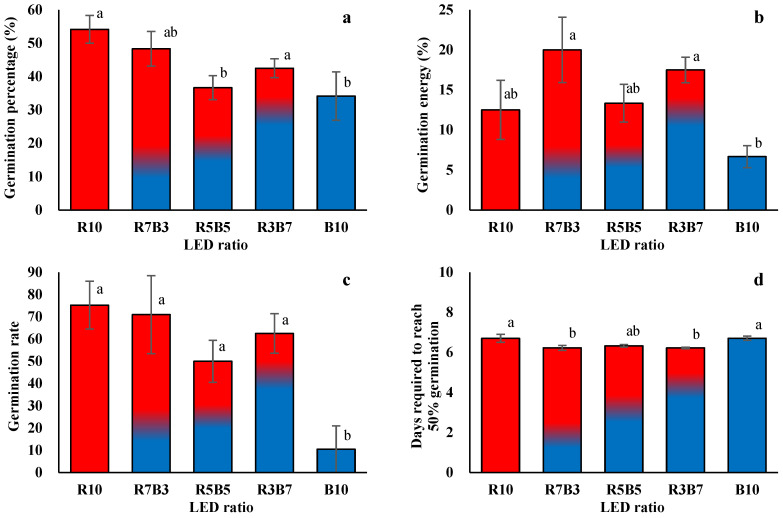
Seed germination characteristics of *Ipomoea aquatica* Forsk. as affected by different light-emitting diode (LED) combinations: GP, germination percentage (**a**); GE, germination energy (**b**); GR, germination rate (**c**); T50, days required to reach 50% germination (**d**). Values represent means ± standard error (SE). Different letters at the top of the bars indicate significance in different light qualities (*p* < 0.05).

**Figure 2:**
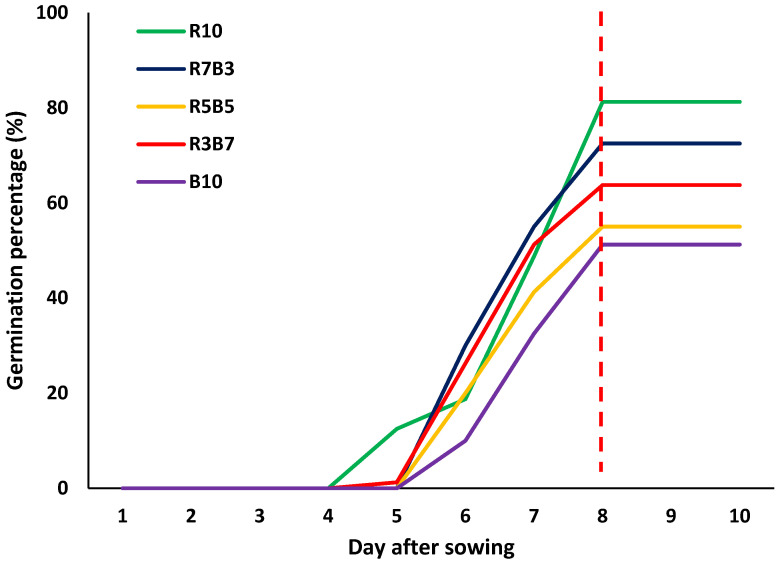
Effects of different combinations of light-emitting diodes (LEDs) on the cumulative germination percentage of *Ipomoea aquatica* Forsk. seedlings. The red dashed line indicates the point at which germination plateaued.

LED spectral composition consistently influenced both the temporal progression and final performance of seed germination in *Ipomoea aquatica* (Figure 1 and Figure 2). Across all treatments, germination began on day 4 after sowing, and most germination events occurred rapidly up to day 7, with little additional increase observed after day 8 (Figure 2). This pattern indicates that, under the present experimental conditions, germination responses were largely completed within a relatively short period. Based on light quality, the responses could be broadly categorized into two distinct patterns. Treatments with a higher proportion of red light (R10, R7B3, and R3B7) exhibited more rapid increases in cumulative germination and achieved higher final germination levels. These treatments also maintained generally higher values for germination vigor indices, including germination energy (GE) and germination rate (GR) (Figure 1a–c). In contrast, the monochromatic blue light treatment (B10) and the equal red–blue ratio treatment (R5B5) showed slower increases in cumulative germination and comparatively lower vigor-related responses. A closer examination of the cumulative germination curves (Figure 2) further highlights these differences. Under red-dominant conditions, a pronounced surge in germination occurred shortly after initiation, followed by earlier stabilization. Conversely, treatments with a higher proportion of blue light displayed slower germination progression and lower final germination levels. These findings suggest that light quality influences not only the final germination percentage but also the rate and synchrony of germination. Although T_50_ differed among treatments (Figure 1d), the overall germination dynamics were consistent with the patterns observed in the cumulative curves and vigor indices. In red-enriched treatments, post-initiation germination progressed more efficiently, whereas the blue-enriched treatment exhibited relatively delayed or constrained responses. Overall, LED spectral composition exerted integrated effects on germination percentage, germination energy, germination rate, and cumulative germination dynamics. Red-dominant conditions promoted faster and more uniform germination responses in *I. aquatica.*

### 2.2. Effects of Light Quality on Early Growth of Ipomoea aquatica

**Figure 3:**
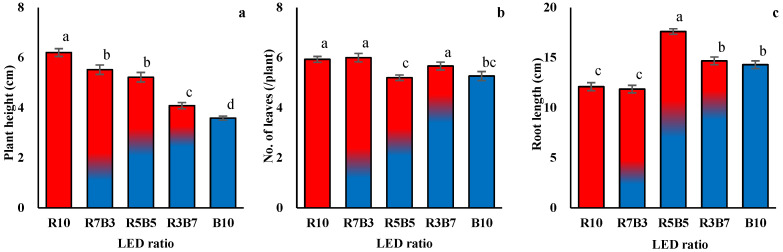
Plant height (**a**), number of leaves (**b**), and root length (**c**) of *Ipomoea aquatica* Forsk. grown under different light qualities with different light-emitting diode (LED) combinations for 20 days. Values represent means ± standard error (SE) (n = 30). Different letters at the top of bars indicate significance in different light qualities (*p* < 0.05).

LED spectral composition induced consistent changes in early growth morphology and biomass allocation patterns in *Ipomoea aquatica* (Table 1, Figure 3). Overall, treatments with a higher proportion of red light enhanced shoot growth, whereas increasing proportions of blue light were associated with a relatively greater contribution of root development. Plant height and shoot biomass were clearly enhanced under red-dominant conditions (Figure 3a; Table 1). This pattern was accompanied by higher shoot-to-root (T/R) ratios, suggesting that red-enriched spectra preferentially promoted shoot elongation and aboveground biomass accumulation. In contrast, treatments with relatively higher blue light proportions exhibited more restrained shoot elongation but comparatively greater allocation to root development (Figure 3c). This response reflects differences in biomass partitioning strategies driven by light quality rather than simple growth suppression. Although variation in leaf number among treatments was relatively modest (Figure 3b), the overall trend was broadly consistent with the patterns observed for shoot elongation and biomass accumulation. Under red-centered conditions, leaf expansion tended to coincide with increased shoot biomass, whereas higher blue light proportions were associated with a greater relative contribution of root growth compared with shoot development. An integrated evaluation of fresh- and dry-weight data (Table 1) further supports these patterns. Red-dominant treatments were characterized by enhanced shoot growth and higher T/R ratios, whereas blue-enriched conditions exhibited a relatively greater proportion of root biomass and dry matter allocation. These findings indicate that LED spectral composition influenced not only total growth but also the direction of biomass allocation between aboveground and belowground organs. In summary, LED light quality simultaneously regulated morphological development and biomass partitioning during early growth. Red-dominant conditions promoted shoot-oriented elongation and biomass accumulation, whereas increasing blue light proportions shifted the balance toward relatively greater root development in *I. aquatica.*

### 2.3. Changes in Maximum Quantum Yield of PSII Under Different Light Qualities

**Figure 4:**
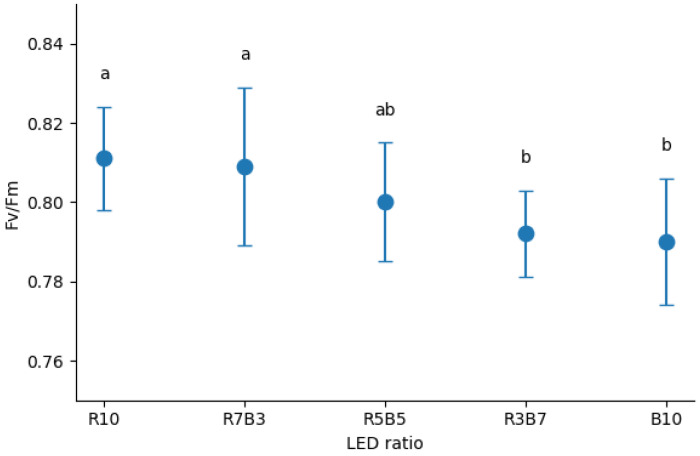
Maximum quantum yield of photosystem II (PSII) (*Fv*/*Fm*) in *Ipomoea aquatica* Forsk. seedlings grown under different light-emitting diode (LED) combinations. Values represent means ± standard error (SE) (n = 30). Different letters above the dots indicate significant differences among treatments according to Duncan’s multiple range test (*p* < 0.05).

LED spectral composition significantly affected the maximum quantum yield of photosystem II (*Fv*/*Fm*) in *Ipomoea aquatica* seedlings (Figure 4). Although the overall range of variation was relatively narrow, consistent trends were observed among the spectral treatments. Treatments with higher proportions of red light maintained comparatively higher and more stable photochemical efficiency, whereas increasing proportions of blue light were associated with a slight reduction in *Fv*/*Fm*. Notably, the relatively elevated *Fv*/*Fm* values observed under red-dominant conditions corresponded with the enhanced shoot growth recorded during the early growth stage, indicating a coordinated response between morphological development and photochemical performance. In contrast, treatments with higher blue light proportions exhibited moderately reduced PSII efficiency, which was consistent with the relatively constrained growth responses observed in the morphological parameters. These results suggest that LED spectral composition influenced not only plant morphology but also the functional status of the photosynthetic apparatus. Although the absolute differences in *Fv*/*Fm* were modest, the consistent and statistically significant variation among treatments indicates that light quality exerts subtle yet meaningful regulatory effects on PSII stability. Overall, red-dominant conditions supported both early growth promotion and the maintenance of photochemical efficiency in *I. aquatica* seedlings.

### 2.4. Quality and Microbiological Evaluation

**Figure 5:**
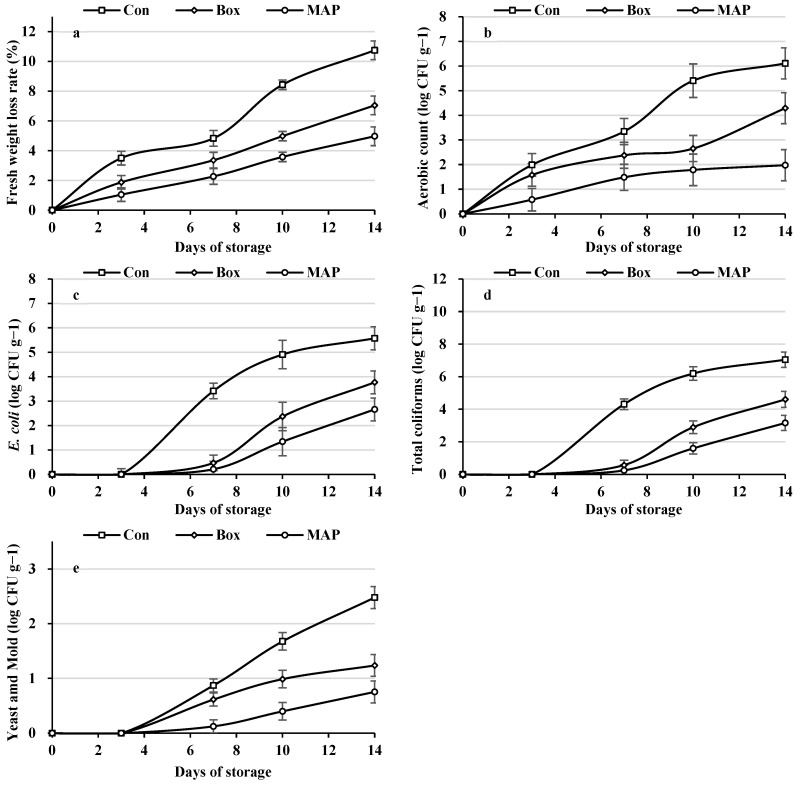
Changes in fresh weight loss rate (**a**), aerobic count (**b**), *Escherichia coli* (**c**), total coliforms (**d**), and yeast and mold counts (**e**) of *Ipomoea aquatica* Forsk. packaged in carton boxes and modified atmosphere packaging (MAP) during 14 days of low-temperature storage after cultivation under R10 LED conditions. Vertical bars represent the standard deviation (SD).

During the 14-day low-temperature storage period, fresh weight loss and all microbiological parameters of *Ipomoea aquatica* (total aerobic count, *E. coli*, total coliforms, and yeast and mold) exhibited a general increasing trend over time (Figure 5; Figures S1–S5). The rate of increase became more pronounced after day 7, indicating that quality deterioration and microbial proliferation progressed in a time-dependent manner. During the initial storage period (0–3 days), several microbiological indicators remained at relatively low levels; however, differences among treatments became progressively more distinct during the mid- to late storage stages. Across all LED conditions, a consistent separation pattern was observed according to the packaging method. However, this separation was not independent of LED treatment; rather, the magnitude of the differences varied depending on the specific combination of LED spectral composition and packaging method. In the non-packaged control, fresh weight loss and microbial growth increased most rapidly throughout the storage period, whereas corrugated box packaging exhibited intermediate suppression effects. In contrast, samples stored under modified atmosphere packaging (MAP) consistently maintained comparatively lower fresh weight loss and reduced microbial levels. This pattern was consistently observed in the Supplementary Figures (Figures S1–S5), indicating that the influence of the packaging method was reproducible across different LED treatments.

Two-way ANOVA results (Table 2) showed that LED treatment (A), packaging method (B), and their interaction (A × B) significantly affected all quality and microbiological parameters (*p* < 0.001). The statistical significance of the interaction term indicates that the effects of the packaging method should not be interpreted independently of the LED conditions. In some LED treatments, microbial growth rates were relatively high; however, even under those conditions, the application of MAP markedly reduced the magnitude of microbial increase. These findings suggest that storage responses were governed not by a single factor alone but by the combined effects of light quality and packaging method. When effect sizes (ηp^2^) were considered (Table 2), packaging method (B) exhibited high explanatory power for most storage-related parameters. At the same time, the A × B interaction also contributed substantially to the total explained variance. Therefore, although the packaging method functioned as an important regulatory factor during storage, its influence should be interpreted within the context of its interaction with LED treatment. In other words, the present results do not indicate that the packaging method is an absolutely dominant single determinant; rather, they demonstrate that it has relatively strong explanatory power under the experimental conditions, while interaction effects remain statistically meaningful. In contrast to the responses observed during the production stage (Section 2.1, Section 2.2 and Section 2.3), where germination, early growth, and PSII efficiency were primarily regulated by LED spectral composition, the storage stage was characterized by the combined and interacting effects of light quality and packaging environment. Thus, whereas light quality played a dominant physiological role during early plant development, storage-related quality maintenance and microbial dynamics were more strongly shaped by interaction-based regulation between preharvest light conditions and postharvest packaging treatments.

## 3. Discussion

This study comprehensively evaluated the effects of LED spectral composition under PFAL conditions on seed germination, early growth, maximum quantum yield of photosystem II (*Fv*/*Fm*), and postharvest quality and microbiological stability of *Ipomoea aquatica*. Across the production stage responses (Section 2.1, Section 2.2 and Section 2.3), a consistent pattern emerged in which red-dominant spectra (or red–blue combinations with a higher proportion of red light) were associated with enhanced germination performance, shoot-oriented early growth, and relatively stable PSII efficiency [11,12,13]. These findings align with previous studies identifying the red-to-blue spectral ratio as a critical regulator of seedling establishment and vegetative development in leafy crops [13,14,15,16].

The promotive effects observed under red-enriched conditions during germination can be interpreted within the framework of photoreceptor-mediated signaling. Red light activates phytochrome by converting the inactive Pr form to the biologically active Pfr form, thereby facilitating germination initiation and early seedling growth [17,18]. Phytochrome signaling is closely linked to hormonal regulation, particularly the modulation of the GA/ABA balance, which governs dormancy release and embryo growth resumption [19]. The enhanced germination synchrony and vigor indices observed under red-dominant treatments in the present study are therefore consistent with established phytochrome-mediated regulatory mechanisms [20]. It should be noted that the baseline germination capacity of the seed lot used in this study was approximately 60%, as provided by the supplier. Across all LED treatments, including the red-dominant conditions, final germination percentages remained within this biological range. This suggests that intrinsic seed quality may have constrained the maximum attainable germination response. Therefore, while LED spectral composition significantly influenced germination dynamics (rate and synchrony), it did not override the inherent viability limitations of the seed lot. The observed spectral effects should thus be interpreted as relative improvements within the physiological constraints of the initial seed quality, rather than absolute enhancement of germination capacity.

During early vegetative growth, the red-dominant spectra were associated with increased shoot elongation, greater shoot biomass accumulation, and higher shoot-to-root ratios. Red light is known to regulate stem elongation and leaf expansion through phytochrome-B-mediated pathways [21], potentially enhancing carbon assimilation and assimilate allocation to aboveground organs. In contrast, increasing blue light proportions were associated with relatively greater root allocation, suggesting a shift in biomass partitioning rather than uniform growth suppression. Blue light, mediated through cryptochrome and phototropin pathways, influences stomatal behavior, chlorophyll biosynthesis, and morphogenetic regulation [22]. The observed redistribution of biomass under blue-enriched conditions likely reflects differential developmental regulation rather than reduced physiological capacity [23].

Although the absolute differences in *Fv*/*Fm* were modest, statistically significant variation among spectral treatments indicates that light quality subtly influenced photochemical efficiency. The relatively stable PSII performance observed under red-dominant treatments corresponded with enhanced shoot growth, suggesting coordinated regulation of morphological development and photosynthetic functionality. These findings are consistent with reports that red–blue spectral ratios influence electron transport stability and photosynthetic efficiency under artificial lighting systems [24,25]. Importantly, the magnitude of *Fv*/*Fm* differences should be interpreted as physiologically meaningful but not indicative of severe photoinhibition or dysfunction.

The growth temperature applied in this study (25 ± 1 °C) represents a commonly used PFAL management condition but may not correspond precisely to the optimal temperature range for *I. aquatica*, a tropical species. Since light-quality responses can interact with temperature conditions [22], it is possible that the magnitude of spectral effects was influenced by the thermal environment. Therefore, the present findings should be interpreted within the specific temperature context of this experiment, and future research examining spectrum × temperature interactions would further refine practical recommendations.

In contrast to the production stage, storage-related quality and microbiological responses were shaped by the combined effects of LED treatment and packaging method. Although the packaging method exhibited relatively high explanatory power across most storage parameters, the presence of significant A × B interaction effects indicates that its influence was not independent of preharvest light conditions. The magnitude of packaging effects varied depending on the LED spectral treatment, demonstrating that postharvest responses were governed by treatment combinations rather than by a single dominant factor.

Modified atmosphere packaging (MAP) regulates internal gas composition, thereby reducing respiration rates and moisture loss, and has been widely recognized as an effective approach for extending the shelf life of fresh produce [26,27,28]. Reduced oxygen availability and elevated carbon dioxide concentrations suppress microbial proliferation and metabolic activity [29]. However, the present results indicate that the effectiveness of packaging strategies must be interpreted within an interaction framework, where preharvest light quality influences subsequent storage behavior.

Effect size analysis (ηp^2^) further supports this interpretation. While the packaging method accounted for a substantial proportion of the explained variance in several storage-related parameters, the interaction between LED treatment and the packaging method also contributed meaningfully to total variance. Accordingly, it would be statistically inappropriate to characterize the packaging method as an absolute primary determinant [30]. Rather, it functioned as a factor with comparatively strong explanatory power under the present experimental conditions, operating within an interaction-based regulatory system.

Taken together, the results demonstrate stage-specific regulatory patterns in PFAL-based production of *I. aquatica*. During the production phase, spectral composition played a dominant physiological role in shaping germination dynamics, biomass allocation, and PSII efficiency. During the storage phase, however, quality maintenance and microbial stability were determined by the interaction between preharvest light conditions and postharvest packaging environment. Therefore, optimization strategies for PFAL systems should adopt a stage-integrated approach: red-enriched spectra to promote uniform and vigorous early growth during production, combined with appropriately designed packaging systems to stabilize quality and microbial status during storage and distribution.

## 4. Materials and Methods

### 4.1. Plant Materials and Growth Conditions

This study was performed in a plant factory system at the Department of Smart Agriculture Convergence, Kangwon National University, Republic of Korea. The experiment was arranged in a completely randomized design (CRD) with five LED light treatments. Each LED treatment was supplied by an independent LED panel, physically separated within the plant factory system to ensure independent light environments. For each LED treatment, four plug trays were used as biological replicates, and each tray was considered one experimental unit for statistical analysis. Seeds of *I. aquatica* Forsk. were sourced from ASIASEED Co., Ltd., (Seoul, Republic of Korea). The baseline germination percentage of the seed lot, as provided by the supplier, was 60%, indicating moderate initial viability. Therefore, spectral treatment effects were evaluated within the biological limits of this seed lot. To ensure seed uniformity, seeds were pre-screened by flotation in distilled water for 10 min, and only fully submerged seeds were selected for sowing. Seeds were soaked in distilled water for 6 h prior to sowing and sown at a density of 30 seeds per tray in 40-cell plug trays filled with a commercial horticultural substrate (Best, Shinsung Mineral Co., Ltd., Seongnam-si, Republic of Korea). Seeds were sown at a uniform depth of approximately 1 cm to ensure consistent germination conditions. Thus, each treatment consisted of four independent trays (n = 4), each containing 30 seeds. After sowing, the plug trays were irrigated with 2 L of distilled water every three days using a bottom-irrigation system. The growing medium included zeolite, perlite, vermiculite, cocopeat, and peat moss, providing the necessary mineral and organic components for plant growth and development. After sowing, the plug trays were irrigated with 2 L of distilled water every three days using a bottom-irrigation system. Throughout the germination and growth period, environmental conditions were maintained at a temperature of 25 ± 1 °C and a relative humidity of 60 ± 10%. The photoperiod was set to 12 h light and 12 h dark. The LED lighting system delivered a photosynthetic photon flux density (PPFD) of approximately 160 μmol m^−2^s^−1^ at the canopy level. No transplanting was performed after germination, and seedlings were grown continuously in the same plug trays and substrate throughout the experimental period. To specifically evaluate the effects of LED spectral treatments without confounding nutritional variables, no additional fertilization was applied during the experiment. Plants were grown for a total of 20 days after sowing, and all growth and physiological measurements were conducted at the end of this cultivation period. To minimize positional effects within each LED treatment area, trays were randomly repositioned daily within their respective LED zones. Because each treatment was supplied by an independent LED panel and trays were treated as independent units, statistical independence among replicates was maintained.

### 4.2. LED Light Treatments

**Figure 6:**
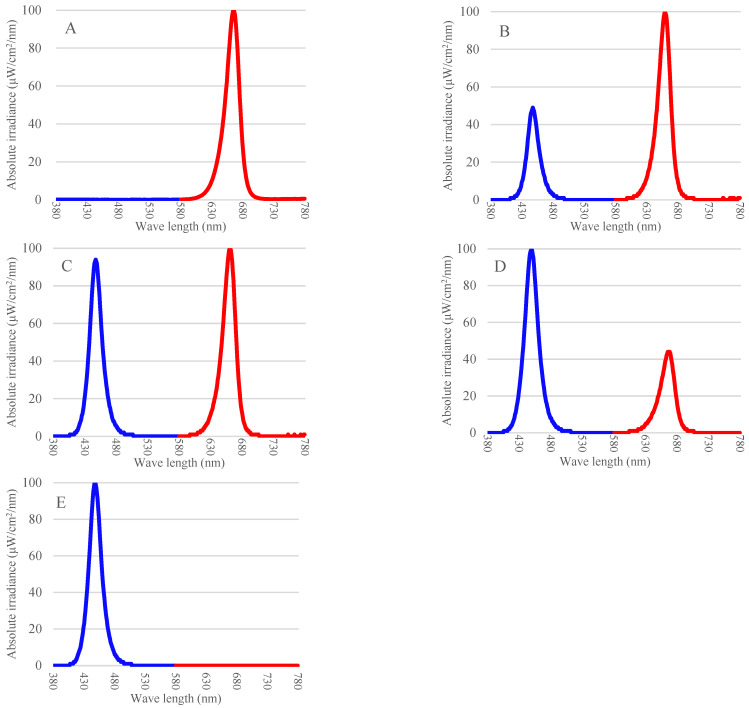
Spectral distribution in various light-emitting diode combinations: (**A**) R10; (**B**) R7B3; (**C**) R5B5; (**D**) R3B7; (**E**) B10.

The LED light sources used in this study included two monochromatic treatments and three combined spectral treatments, all custom-manufactured by Bissol LED (Seoul, Republic of Korea). The monochromatic treatments were 100% red light (650 nm) and 100% blue light (450 nm). The combined light treatments featured red and blue light mixed in ratios of 7:3 (R7B3), 5:5 (R5B5), and 3:7 (R3B7) (Figure 6).

### 4.3. Assessment of Germination Characteristics Under LED Light

Seed germination was monitored daily for 10 days after sowing. A seed was considered germinated when the seedling emerged more than 1 mm above the substrate surface. Germination characteristics were evaluated based on cumulative germination data collected during the monitoring period.

Germination percentage (GP) was calculated according to Kim et al. (2022) [31] as follows:

GP (%) = (No. of germinated seeds/Total number of seeds) × 100(1)

Germination energy (GE) was calculated following Kim et al. (2022) [31]:

GE (%) = (T/2)/S × 100(2)

where T is the total number of irradiation days, and S is the total number of germinated seeds.

Germination rate (GR) was calculated according to Bartlett (1937) [32]:

GR = a + (a + b) + (a + b+ c) + … + (a + b + c + … + m)/n (a + b + c + … + m)(3)

where a, b, and c represent the number of seedlings counted at the first, second, and third observations, respectively; n is the total number of counts; and m is the number of seedlings at the final count.

T_50_ was calculated according to the following equation (Coolbear et al., 1984) [33]:

T_50_ (days) = T_i_ + (T_j_ − T_i_) × (N/2 − N_i_)/(N_j_ − N_i_) (4)

where N is the final number of germinated seeds; N_i_ and N_j_ are the cumulative numbers of germinated seeds at times T_i_ and T_j_, respectively; and N_i_ < N/2 < N_j_.

### 4.4. Measurement of Growth Characteristics Under LED Light

Growth parameters were evaluated 20 days after germination. The seedlings used for growth and physiological measurements originated from the same germination experiment described above. For growth measurements, thirty seedlings per treatment were randomly selected from the four replicate trays established for the germination experiment (approximately 7–8 seedlings per tray). Thus, a total of 120 seeds per treatment (30 seeds × 4 trays) were initially used for germination assessment. To ensure statistical independence and avoid pseudoreplication, mean values were first calculated at the tray level, and these tray means (n = 4) were used as the experimental units for statistical analysis. The measured parameters included plant height, number of leaves, leaf length, leaf width, and root length, following the methods outlined by Kim and Kang (2025) [34]. Fresh weight was separately recorded for the shoot (including leaf sheath) and root (including seed remnants and roots) using an electronic balance (WBA-220, Wisd, Wertheim, Germany). Leaf area was analyzed with Easy Leaf Area software (original 2014 release; University of California, Davis, CA, USA) (Easlon and Bloom, 2014) [35], adhering to the procedure described by Kim et al. (2022) [36]. Dry weight was determined by drying the samples at 70 °C for 72 h in an incubator (HK-BI025, Hankuk S&I, Anyang-si, Republic of Korea). Plant height and root length were measured directly with a ruler.

### 4.5. Measurement of the Maximum Quantum Yield of Photosystem II (Fv/Fm)

The maximum quantum yield of photosystem II (*Fv*/*Fm*) was measured on the first fully expanded leaf of 30 seedlings per treatment at 20 days after germination. Chlorophyll fluorescence was recorded at 06:00 using a Junior-PAM chlorophyll fluorometer (Heinz Walz GmbH, Effeltrich, Germany). Before measurement, the plants were dark-adapted for 20 min and then exposed to a saturating light pulse (445 nm, 2800 μmol m^−2^ s^−1^). Minimum fluorescence (*Fo*), maximum fluorescence (*Fm*), and variable fluorescence (*Fv* = *Fm* − *Fo*) were recorded, and *Fv*/*Fm* was calculated as (*Fm* − *Fo*)/*Fm*.

### 4.6. Quality Evaluation and Microbiological Analysis

Quality and microbiological analyses were performed on plants harvested 20 days after germination from each LED treatment. The storage experiment was arranged in a completely randomized design with LED treatment and packaging method as fixed factors. For each LED treatment, samples were randomly allocated to packaging treatments. Each independently sealed container was considered one experimental unit. For each LED × packaging combination, four independent packages were prepared (n = 4). Each package contained 100 g of fresh seedlings. The samples were packaged either in conventional corrugated cardboard boxes or in MAP and stored at 8 ± 1 °C for 14 days. For the MAP, a polypropylene film (50 μm thickness) was laser-perforated to achieve an oxygen transmission rate (OTR) of 40,000 cc·m^−2^·day^−1^·atm^−1^ (Daeryung Packaging Industry, Busan, Republic of Korea).

Samples were sealed in containers (123 × 175 × 90 mm) using an impulse heat sealer (SC200-IP, KumKang, Seoul, Republic of Korea). Weight loss was calculated as the percentage difference between the initial and final sample weights. Microbiological analyses were conducted in five replicates following the methods described by Choi et al. (2021, 2022) [37,38]. For each sample, 2 g (dry weight) was mixed with 18 mL of sterile distilled water in a sterile stomacher bag and homogenized for 3 min using a stomacher (Power Mixer, B&F Korea, Gimpo-si, Republic of Korea). The homogenate (0.2 mL) was diluted with 19.8 mL of sterile water to achieve a 1000-fold dilution. Aliquots (1.0 mL) were plated onto Petrifilm™ count plates (3M Microbiology Products, St. Paul, MI, USA). Aerobic bacteria were incubated at 35 °C for 48 h, *E*. *coli* at 35 °C for 24 h, and yeasts and molds at 25 °C for 72 h. Colony counts were determined using an automated plate reader (Petrifilm Plate Reader, 3M, St. Paul, MI, USA) and expressed as log CFU g^−1^.

### 4.7. Statistical Analysis

Statistical analyses were performed using SPSS software (version 18.0; SPSS Inc., Chicago, IL, USA). For germination and growth parameters, data were analyzed using one-way ANOVA under a completely randomized design, with LED treatment as the main factor. In these analyses, the plug tray was considered the experimental unit (n = 4). For storage and microbiological data, a two-way ANOVA was conducted with LED light treatment and packaging method as fixed factors. In this case, each independently prepared package container was treated as the experimental unit. Mean comparisons among treatments were performed using Duncan’s multiple range test (*p* < 0.05). Because multiple comparisons were conducted, interpretation focused primarily on overall ANOVA results to avoid overemphasis of pairwise differences.

## 5. Conclusions

This study examined how LED light quality influences the early growth, physiological stability, and postharvest responses of *Ipomoea aquatica* Forsk. grown in a plant factory system. Red-dominant LED treatments (R10 and R7B3) were associated with improved germination uniformity and enhanced early shoot growth while maintaining a relatively stable maximum quantum yield of photosystem II (*Fv*/*Fm*). These responses suggest that red-enriched spectra may support efficient early-stage development under the experimental conditions applied. In contrast, postharvest quality parameters and microbiological dynamics were strongly influenced by packaging method, with significant interaction effects observed between LED treatment and packaging condition. Modified atmosphere packaging (MAP) was consistently associated with reduced fresh weight loss and lower microbial counts during storage; however, its effects should be interpreted within the context of its interaction with preharvest light conditions. Overall, the findings indicate stage-specific regulatory patterns in PFAL-based production of *I. aquatica*, in which spectral composition primarily influenced production-stage responses, whereas storage outcomes were governed by the combined effects of light quality and packaging environment. These results support a stage-integrated management approach, in which red-enriched spectra may be applied during early growth and appropriate packaging strategies are implemented during storage to promote production stability and postharvest quality maintenance.

## Figures and Tables

**Table 1:** Growth characteristics of *Ipomoea aquatica* Forsk. grown under different light-emitting diode (LED) combinations.

Treatment	Leaf Length (cm)	Leaf Width (cm)	Leaf Area (cm^2^/Plant)	Fresh Weight (g/Plant)		Dry Weight (g/Plant)		T/R Ratio
				Shoot	Root	Shoot	Root	
R10	6.44 a ^z^	3.80 a	18.37 a	4.615 a	0.658 c	0.283 a	0.043 c	7.28 a
R7B3	5.60 b	2.79 b	11.74 c	4.023 b	1.038 b	0.236 c	0.039 c	7.02 a
R5B5	5.13 c	2.84 b	10.93 c	3.362 c	1.028 b	0.199 d	0.059 b	3.52 bc
R3B7	5.18 c	3.11 a	12.10 bc	3.946 b	1.217 a	0.232 c	0.131 a	1.80 c
B10	5.39 bc	3.23 a	13.04 b	4.254 b	1.099 ab	0.262 b	0.069 b	4.54 b

^z^ Values are means ± standard error. Means followed by different letters within the same column are significantly different according to Duncan’s multiple range test (*p* < 0.05).

**Table 2:** The fresh weight loss rate, aerobic count, *Escherichia coli*, total coliforms, and yeast and mold of *Ipomoea aquatica* Forsk. packed in carton boxes and modified atmosphere packaging (MAP) after low-temperature treatment and stored for 14 days.

Source	Fresh Weight Loss Rate (%)			Aerobic Count (log CFU g^−1^)		
	*F* Value	*p* Value	ηp^2^	*F* Value	*p* Value	ηp^2^
LED treatment (A)	1004.897	<0.001	0.950	119.669	<0.001	0.695
Packed method (B)	758,845.634	<0.001	0.999	23,897.984	<0.001	0.996
A × B	1785.559	<0.001	0.986	57.992	<0.001	0.688
Source	*E. coli* (log CFU g^−1^)			Total coliforms (log CFU g^−1^)		
	*F* value	*p* value	ηp^2^	*F* value	*p* value	ηp^2^
LED treatment (A)	232.144	<0.001	0.816	363.602	<0.001	0.874
Packed method (B)	9622.057	<0.001	0.989	10,023.509	<0.001	0.990
A × B	191.011	<0.001	0.879	194.005	<0.001	0.881
Source		Yeast and Mold (log CFU g^−1^)				
	*F* value		*p* value		ηp^2^	
LED treatment (A)	236.132		<0.001		0.818	
Packed method (B)	41,428.343		<0.001		0.997	
A × B	46.163		<0.001		0.638	

## Data Availability

The original contributions presented in this study are included in the article/Supplementary Materials. Further inquiries can be directed to the corresponding authors.

## Supplementary Materials

The following supporting information can be downloaded at https://www.mdpi.com/article/10.3390/plants15050776/s1. Figure S1: Changes in fresh weight loss rate of *Ipomoea aquatica* Forsk. cultivated under different LED light quality conditions [R7B3 (a), R5B5 (b), R3B7 (c), and B10 (d)] and packaged in carton boxes and modified atmosphere packaging (MAP) during 14 days of low-temperature storage. Vertical bars represent standard deviation (SD); Figure S2: Changes in aerobic count of *Ipomoea aquatica* Forsk. cultivated under different LED light quality conditions [R7B3 (a), R5B5 (b), R3B7 (c), and B10 (d)] and packaged in carton boxes and modified atmosphere packaging (MAP) during 14 days of low-temperature storage. Vertical bars represent standard deviation (SD); Figure S3: Changes in Escherichia coli counts of *Ipomoea aquatica* Forsk. cultivated under different LED light quality conditions [R7B3 (a), R5B5 (b), R3B7 (c), and B10 (d)] and packaged in carton boxes and modified atmosphere packaging (MAP) during 14 days of low-temperature storage. Vertical bars represent standard deviation (SD); Figure S4: Changes in total coliform counts of *Ipomoea aquatica* Forsk. cultivated under different LED light quality conditions [R7B3 (a), R5B5 (b), R3B7 (c), and B10 (d)] and packaged in carton boxes and modified atmosphere packaging (MAP) during 14 days of low-temperature storage. Vertical bars represent standard deviation (SD); Figure S5: Changes in yeast and mold counts of *Ipomoea aquatica* Forsk. cultivated under different LED light quality conditions [R7B3 (a), R5B5 (b), R3B7 (c), and B10 (d)] and packaged in carton boxes and modified atmosphere packaging (MAP) during 14 days of low-temperature storage. Vertical bars represent standard deviation (SD); Table S1: The fresh weight loss rate, aerobic count, Escherichia coli, total coliforms, and yeast and mold of *Ipomoea aquatica* Forsk. packed in carton boxes and modified atmosphere packaging (MAP) after low-temperature treatment and stored for 14 days.
