# Supplementary material for: Effects of LED Light Combinations on the Growth and Storage Stability of Ipomoea aquatica in a Plant Factory System
Source: Plants (Basel). 2026 Mar 3;15(5):776. doi: 10.3390/plants15050776 (PMC12987022; doi:10.3390/plants15050776)
Supplement: Supplementary file 1 [file plants-15-00776-s001.zip › plants-4172292-supplementary.pdf]

Supplementary Table

Table S1. The fresh weight loss rate, aerobic count, *Escherichia coli*, total coliforms, and yeast and mold of *Ipomoea aquatica* Forsk. packed in carton boxes and modified atmosphere packaging (MAP) after low-temperature treatment and stored for 14 days.

LED treatment	Packaging	Fresh weight loss rate (%)	Aerobic count (log CFU g^{-1})	*E.coli* (log CFU g^{-1})	Total coliforms (log CFU g^{-1})	Yeast and Mold (log CFU g^{-1})
R10	Con	10.75 e[z]	6.11 d	5.57 e	7.05 d	2.48 d
	Box	7.05 i	4.29 h	3.77 h	4.61 i	1.24 f
	MAP	4.97 l	1.98 l	2.66 j	3.16 kl	0.75 i
R7B3	Con	10.89 d	6.73 b	5.73 d	7.43 c	2.73 c
	Box	7.29 h	4.61 f	3.88 h	4.94 h	1.24 f
	MAP	5.10 j	2.37 j	2.70 ij	3.30 k	0.76 i
R5B5	Con	11.10 c	6.88 a	8.01 a	10.84 a	2.73 c
	Box	7.61 g	4.42 g	4.60 f	6.01 e	1.24 f
	MAP	5.00 k	1.95 l	2.29 k	2.98 m	0.74 i
R3B7	Con	11.50 b	6.73 b	6.27 b	8.82 b	2.80 b
	Box	7.61 g	4.61 f	4.29 g	5.81 f	1.24 f
	MAP	4.74 m	2.28 k	2.31 k	3.05 m	0.85 h
B10	Con	11.70 a	6.36 c	6.04 c	8.68 b	2.90 a
	Box	7.65 f	4.81 e	3.77 h	5.30 g	1.48 e
	MAP	4.41 n	2.66 i	2.79 i	3.84 j	0.88 g
				ANOVA [y]		
LED treatment (A)		***	***	***	***	***
Packaging (B)		***	***	***	***	***

A × B	***	***	***	***	***

[z] Within each column, the mean values followed by different letters are significantly different according to Duncan's multiple range test ($p < 0.05$).

[y] *, **, *** Significant at the 0.05, 0.01 and 0.001 probability level, respectively. NS = Not significant.

Supplementary Figure

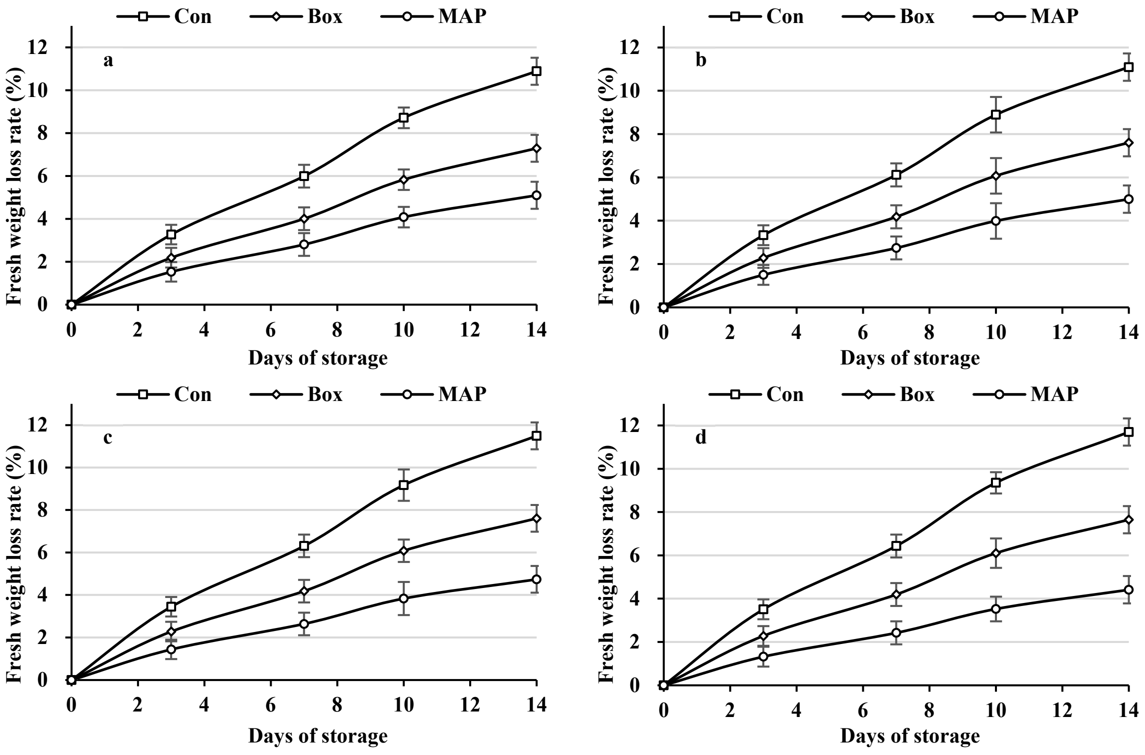

Figure S1. Changes in fresh weight loss rate of *Ipomoea aquatica* Forsk. cultivated under different LED light quality conditions [R7B3 (a), R5B5 (b), R3B7 (c), and B10 (d)] and packaged in carton boxes and modified atmosphere packaging (MAP) during 14 days of low-temperature storage. Vertical bars represent standard deviation (SD).

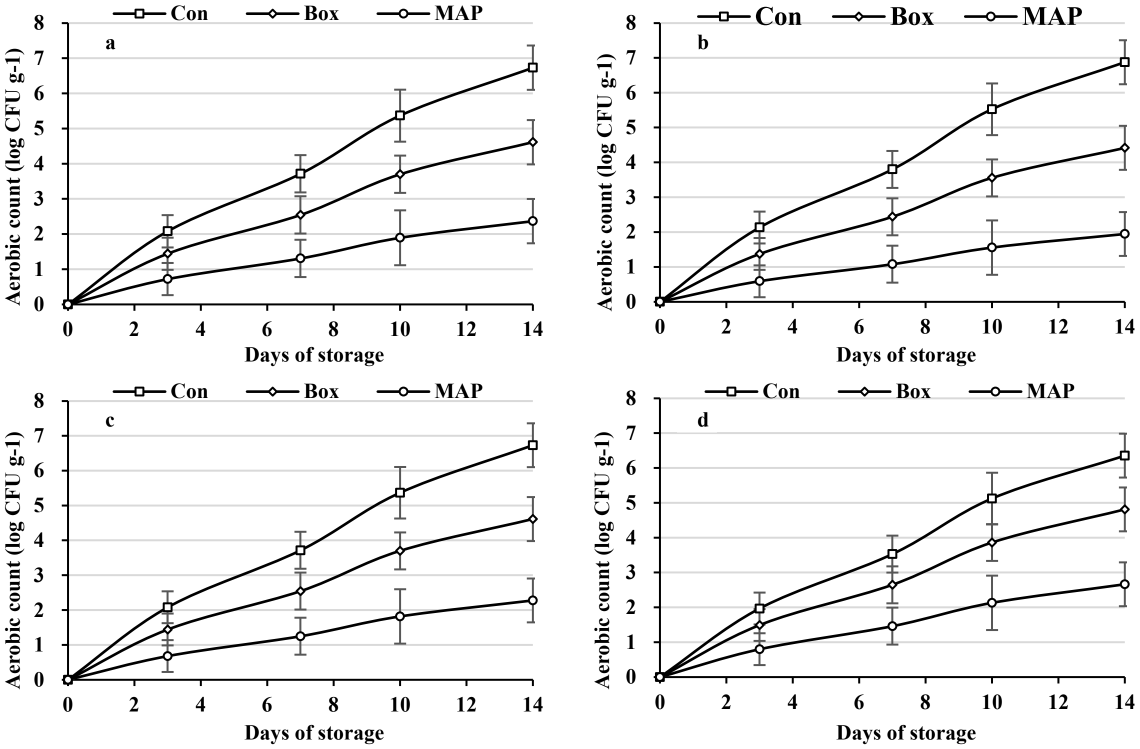

Figure S2. Changes in aerobic count of *Ipomoea aquatica* Forsk. cultivated under different LED light quality conditions [R7B3 (a), R5B5 (b), R3B7 (c), and B10 (d)] and packaged in carton boxes and modified atmosphere packaging (MAP) during 14 days of low-temperature storage. Vertical bars represent standard deviation (SD).

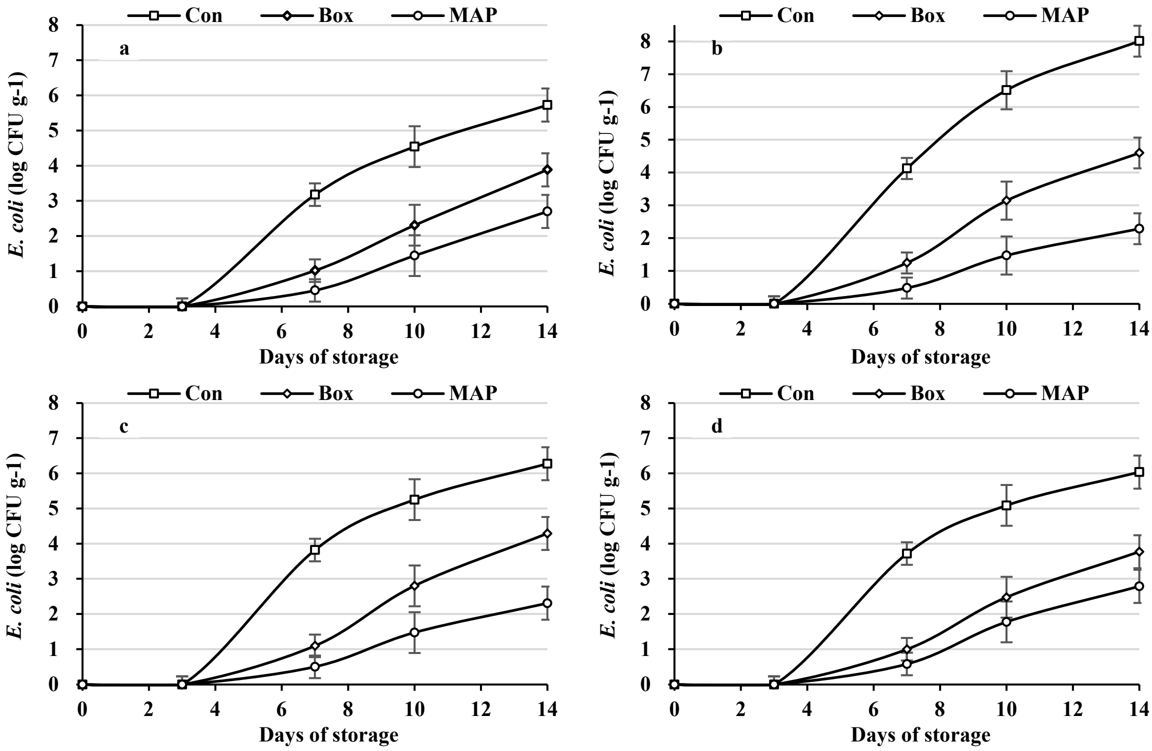

Figure S3. Changes in *Escherichia coli* counts of *Ipomoea aquatica* Forsk. cultivated under different LED light quality conditions [R7B3 (a), R5B5 (b), R3B7 (c), and B10 (d)] and packaged in carton boxes and modified atmosphere packaging (MAP) during 14 days of low-temperature storage. Vertical bars represent standard deviation (SD).

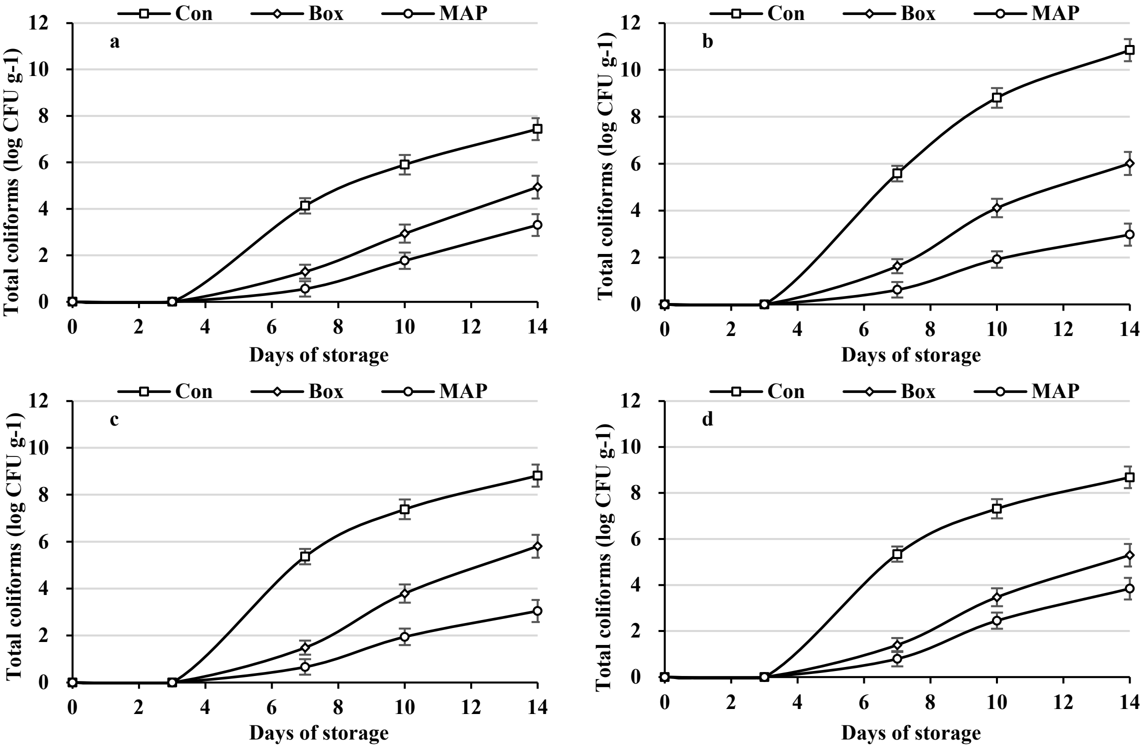

Figure S4. Changes in total coliform counts of *Ipomoea aquatica* Forsk. cultivated under different LED light quality conditions [R7B3 (a), R5B5 (b), R3B7 (c), and B10 (d)] and packaged in carton boxes and modified atmosphere packaging (MAP) during 14 days of low-temperature storage. Vertical bars represent standard deviation (SD).

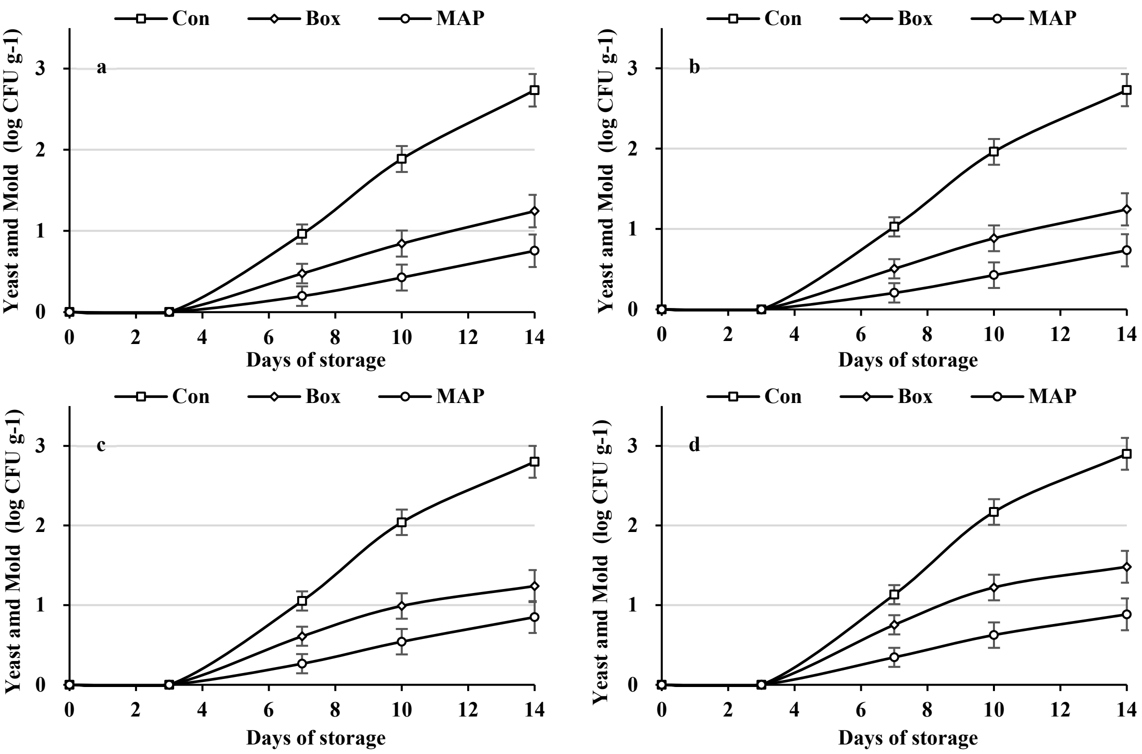

Figure S5. Changes in yeast and mold counts of *Ipomoea aquatica* Forsk. cultivated under different LED light quality conditions [R7B3 (a), R5B5 (b), R3B7 (c), and B10 (d)] and packaged in carton boxes and modified atmosphere packaging (MAP) during 14 days of low-temperature storage. Vertical bars represent standard deviation (SD).
